# An optimization model to prioritize fuel treatments within a landscape fuel break network

**DOI:** 10.1371/journal.pone.0313591

**Published:** 2024-12-17

**Authors:** Dung Nguyen, Yu Wei, Erin J. Belval, Matthew P. Thompson, Benjamin M. Gannon, Jesse D. Young, Christopher D. O’Connor, David E. Calkin

**Affiliations:** 1 Department of Forest and Rangeland Stewardship, Colorado State University, Fort Collins, Colorado, United States of America; 2 USDA Forest Service, Rocky Mountain Research Station, Fort Collins, Colorado, United States of America; 3 Pyrologix LLC, Missoula, Montana, United States of America; 4 USDA Forest Service, National Office, Fire and Aviation Management, Fort Collins, Colorado, United States of America; 5 USDA Forest Service, Rocky Mountain Research Station, Missoula, Montana, United States of America; Marshall University, UNITED STATES OF AMERICA

## Abstract

We present a mixed integer programming model for prioritizing fuel treatments within a landscape fuel break network to maximize protection against wildfires, measured by the total fire size reduction or the sum of Wildland Urban Interface areas avoided from burning. This model uses a large dataset of simulated wildfires in a large landscape to inform fuel break treatment decisions. Its mathematical formulation is concise and computationally efficient, allowing for customization and expansion to address more complex and challenging fuel break management problems in diverse landscapes. We constructed test cases for Southern California of the United States to understand model outcomes across a wide range of fire and fuel management scenarios. Results suggest optimal fuel treatment layouts within the Southern California’s fuel break network responding to various model assumptions, which offer insights for regional fuel break planning. Comparative tests between the proposed optimization model and a rule-based simulation approach indicate that the optimization model can provide significantly better solutions within reasonable solving times, highlighting its potential to support fuel break management and planning decisions.

## 1. Introduction

The escalation of wildland fire activity has raised significant concerns throughout the world. The United States (US), for instance, has experienced pressing wildfire issues with longer fire seasons, increased fire frequency, larger fire sizes, and higher levels of fire damage over the past five decades [1–3], leading to the country’s surging expenditures on wildfire prevention, suppression, and other fire adaptation programs [1, 4–8]. With global wildfire activity predicted to rise in the next century [9], wildfire concerns are likely to persist and effective management of wildfires will remain critical worldwide.

One major emphasis of pre-fire mitigation is managing wildland fuels, which accumulate over time and contribute to escalating wildfire hazards [10, 11]. For example, excessive fuel build-up has put 230 million acres of US federal lands at moderate-to-high risk from wildfires [3], driving the development of the 2022 National Strategy for fuel reduction in 50 million acres of land over the next ten years [8]. Wildland fuels can be reduced by vegetation treatments (as known as fuel treatments) such as tree thinning, prescribed burning, herbicides, and other mechanical and chemical methods [12–15]. Past studies have shown that in both fire-prone wildlands and wildland urban interface areas, fuel treatments can be effective e in slowing fire spread [16], reducing fire size [17–19], decreasing fire intensity and severity [20, 21], and facilitating timely suppression responses and firefighter safeguards [22, 23]. Two common design patterns of fuel treatments within a landscape are area-wide treatments and linear fuel breaks. Area-wide fuel treatments typically focus on reducing fuel loads in wide blocks of land to decrease fire intensity and severity, while linear fuel breaks reduce or alter the arrangement of fuels within narrow strips of land to assist in wildfire suppression [24, 25]. Fuel breaks are often constructed around a fire break, which is a narrower feature with complete fuel removal to act as a barrier to surface fire spread. Despite ongoing debates about the relative merits between these two approaches [26], linear fuel breaks continue to draw interest for wildfire management in various countries, such as the United States [27, 28], Portugal [29], China [30], Belarus and Ukraine [31].

The strategic placement of fuel breaks is essential for their effectiveness in supporting wildfire suppression. Ideal locations for fuel breaks are areas that offer advantages for fire control and containment activities, such as easy access for firefighting crews, proximity to roads, or the presence of natural barriers like rivers, ridges, or sparsely vegetated terrain that can help slow fire progression. Fuel breaks are often established as a network of interconnected linear segments across the landscape to prevent fires from breaching the gaps among fuel breaks. Designing and maintaining a fuel break network (FBN) is often a complex process that requires both local expertise and spatial analyses of fire spread patterns and fire control opportunities [24, 29, 30, 32, 33]. Modeling techniques, including simulation and optimization, can aid in evaluating FBN designs. Simulation typically examines a limited set of predefined scenarios, while optimization explores the entire feasible solution space to identify the most efficient option that meets management objectives. Several comprehensive reviews indicate that fuel treatment modeling research is heavily dominated by simulation [34, 35]. Optimization models are less common and often confined to demonstrating modeling concepts with limited real-world applicability [36], likely due to model complexity and time-consuming computation that inhibit their capability to provide scalable solutions for larger, more complex landscapes. Moreover, most existing models focus on area-wide fuel treatments, with little attention to address the fuel treatment problem from an FBN perspective.

Although there are numerous modeling studies on fuel treatment management, few of them specifically focus on treating FBNs. One option to manage an FBN is to treat all fuel breaks within the network. Oliveira et al. found that more intense treatments across the entire network led to greater reductions in both fire size and burn probability [37]. Similarly, Zong et al. showed that treating an entire FBN with specific configurations of fuel break densities, widths, and spatial locations may significantly decrease burn probability across a landscape.

Treating an entire FBN, especially a large one, may be challenging due to cost and time constraints. This is because fuel treatments are expensive [4, 38–40], and they often require periodic retreatments to maintain their effectiveness over time [41, 42]. Consequently, recent modeling research has shifted their focus to prioritizing limited fuel treatments within an FBN. For example, Belavenutti et al. [27] developed a model to prioritize discrete projects (i.e., sub-networks within an FBN) for implementing fuel treatments over a 10 to 20-year time frame. They designed 13 scenarios where the sequence of projects in each scenario was optimized to achieve multiple fire management objectives. This work was extended by Ager et al. [43] to further analyze the tradeoffs between two alternative treatment strategies within each prioritized project, where fuel treatments were implemented in a set of fuel breaks that form either a long linear or a “radical” (i.e., clustered) spatial pattern. Similarly, Aparício et al. [29] developed a simulation-based model to partition fuel treatments within an FBN into a sequence of annual treatments over a 5-year period. They designed 11 scenarios to prioritize the sequence of annual treatments according to their contributions to various fire management goals. All these studies share a similar approach of prioritizing sequential fuel treatments over time, which collectively select a new sub-set of fuel breaks to treat at each time step until treatments cover the entire FBN. While sequential fuel treatments may provide opportunities for budget preparation during the time gap between treatments, ensuring a sufficient treatment budget at a discrete point of time may remain uncertain in real-world scenarios. The budget limitation constraint has been considered in previous modeling studies for scheduling area-wide fuel treatments [44–48]. However, it has not received adequate attention from existing FBN modeling studies.

In this study, we integrate a large set of simulated fires into an optimization model for prioritizing fuel treatments within an FBN to maximize its protection against those wildfires, while considering the limitations imposed by budget constraints. The model evaluates the relative importance of treating each fuel break in the FBN, enabling spatial coordination of treatments to enhance fire containment. Our model possesses two key attributes that facilitate its application: 1) the capability to address landscape-scale fuel break treatment and planning problems for prioritizing the maintenance or expansion of existing FBNs; and 2) a simple but flexible mixed integer programming (MIP) model formulation that can be customized and extended to adapt to various fire management objectives and implementation requirements of diverse landscapes. We tested this model in the Southern California region of the US to gain insights into how fuel break maintenance under different investment scenarios can affect fire management outcomes. Solutions for these test cases were visualized in an Earth Engine web-application, available at https://thumit.users.earthengine.app/view/landscape-fuelbreak-prioritization.

## 2. Materials and methods

### 2.1. Problem description

Our problem considers the need to strategically invest in fuel treatments within an FBN. Given a specific budget, our primary objective is to prioritize treating a subset of existing or candidate fuel breaks in the network to maximize the protective capacity of the FBN, measured by the total reduction in fire size or the sum of Wildland Urban Interface (WUI) areas avoided from burning. Fire suppressions along the treated fuel breaks are assumed effective in stopping fire spread at certain intensities (or flame lengths). The coordination of treated fuel breaks and associated suppressions creates opportunities for containing fires. We designed an optimization model that can configure and evaluate multiple treatment strategies, each represents a different feasible subset of fuel breaks that can be selected, to identify the optimal solution representing the best subset of fuel breaks for prioritizing treatment across the landscape.

We used a large set of simulated future wildfires (more details will be described in the test cases) to guide fuel break selection decisions across the landscape. For each simulated fire, we evaluated the feasibility for its containment by assessing the spatial interaction between the fire’s simulated footprint and the fuel breaks. Containment success was determined based on the treatments applied to the fuel breaks and the effectiveness of fire suppression efforts. The outcomes of containment, such as reductions in total fire size or the protection of WUI areas, were aggregated across all simulated fires. These aggregated metrics were used by our model to optimize the selection of fuel breaks for treatment across the landscape.

To determine the feasibility of fire containment, we employed an approach similar to Aparício et al. [29] to intersect fire footprints with fuel breaks in an FBN (more details in S1 Appendix). In this approach, when a fire can be split by a set of fuel breaks into multiple polygons, the smallest polygon enclosing the fire’s ignition point (also called the “ignition polygon”) is identified. We built upon the ignition polygon approach by further considering two additional conditions to warrant effective fire containment by the ignition polygon:

1. All fuel breaks constituting the ignition polygon must be treated or maintained up to a standard to allow for fire suppression activities.
2. The maximum fire intensity along fuel breaks that constitute the ignition polygon must not exceed a certain threshold to ensure successful fire suppression. This can be customized to consider factors other than fire intensity, such as topography or weather, that may also influence suppression effectiveness.

We focus on providing opportunities to contain fires before they escape from the ignition polygons to address the increasing need to developing safe and effective response strategies to manage large fires [49]. Incorporating the above two conditions helps assess fire containment in a more realistic context of wildfire management that accounts for the effects from both fuel treatment and fire suppression. Our assumption that fire suppression activities are more effective along treated fuel breaks is consistent with evidence from past empirical research [23, 50, 51]. Other empirical studies also indicate that suppressing a fire along treated fuel breaks may not always successfully halt fire spread, especially in cases of high-intensity fires [28, 51]. By assuming successful suppression only when fire intensity (or flame length) falls below a predefined controllable threshold, we aimed to not overestimate suppression effectiveness.

When either of the two specified conditions is unsatisfied for a fire, we assume the fire would grow into its originally simulated footprint. Conversely, when both conditions are met, we assume that a portion of the fire footprint would be contained within its ignition polygon, while the remaining fire footprint portion outside of the ignition polygon would be protected. A fire management objective can be set up to maximize the total protected area aggregated across all fires or maximize the sum of the quantifiable values within the protected area, such as timber volume or WUI area avoided from burning.

### 2.2. Model formulation

We developed a mixed integer program (MIP) to prioritize strategic investment in a landscape’s FBN to maximize the total value protected from wildfires. This MIP focuses on modelling the spatial coordination of fuel treatments within the FBN. In this model, each potential fuel break serves as a treatment decision unit. Given a limited fuel treatment budget, the challenge lies in deciding which fuel breaks within the FBN to treat. Treated fuel breaks are assumed to facilitate fire suppression along these breaks, providing opportunities for effective fire containment under a predefined fire behavior condition (e.g., less extreme fires). It is important to note that this model does not account for fire suppression details such as suppression resources, cost, and time. Although the MIP model is relatively simple compared to many other fuel treatment optimization models, complex spatial analysis is required to identify the spatial relationships between fire and fuel breaks and define model parameters.


$$\text{Maximize}:\sum_{j}V_{j}y_{j}$$ (1)

Subject to:

$$\sum_{i}C_{i}^{F}x_{i}\leq B$$ (2)

$$y_{j}\leq \frac{1}{M_{j}}\sum_{i\in S_{j}^{F}\neq \emptyset }x_{i}\;\forall j$$ (3)

**Fig 1:**
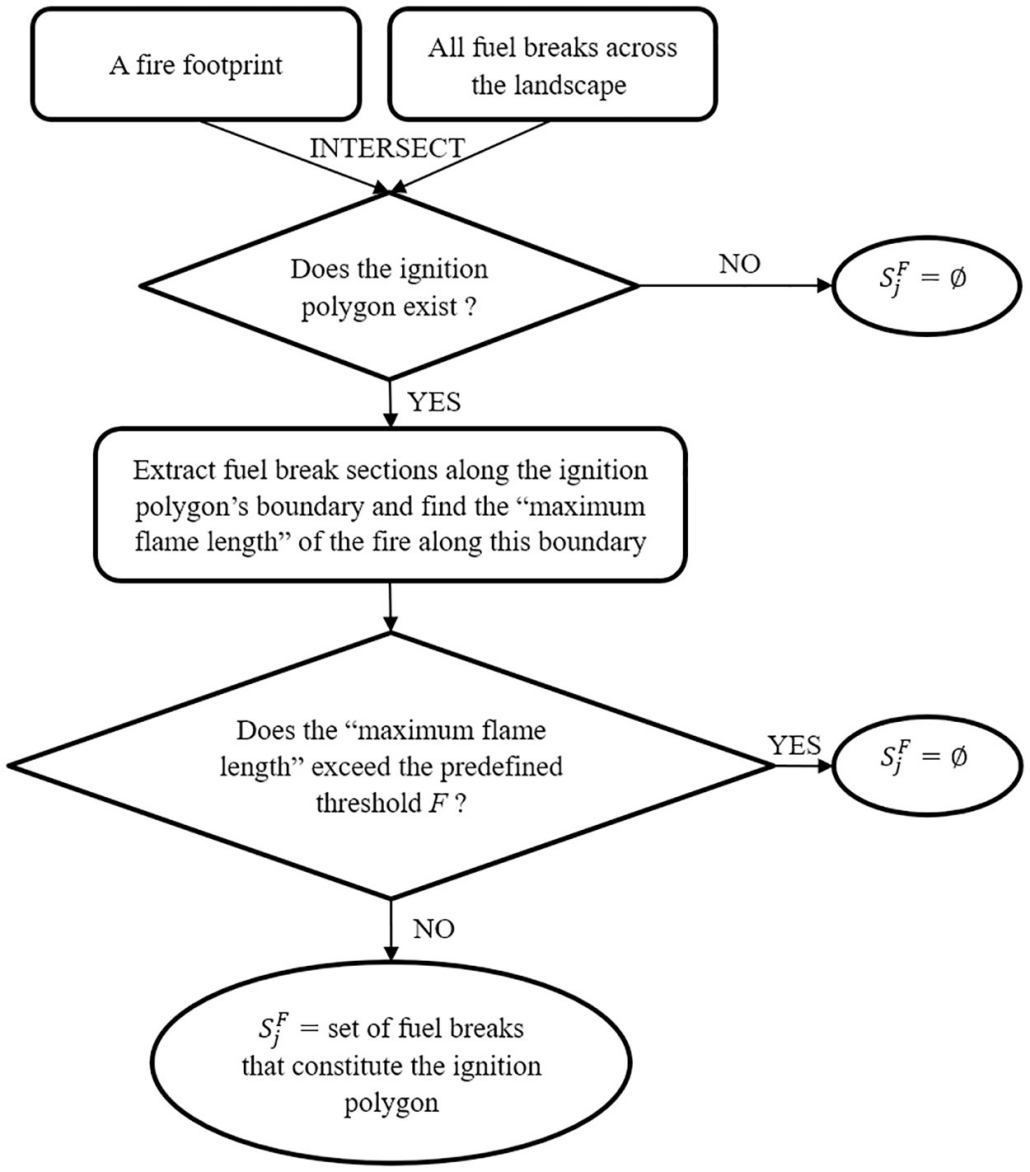
A schematic illustration of how to derive the set of fuel breaks that constitutes a fire’s ignition polygon.

Where:

- *i* is index of a fuel break.
- *j* is index of a wildfire footprint.
- *x*_*i*_ is binary variable: 1 if fuel break *i* is selected for investment.
- *y*_*j*_ is binary variable: 1 if fire *j* is effectively contained within the ignition polygon.
- *M*_*j*_ is the number of breaks that defines the “ignition polygon” of fire *j*.
- *V*_*j*_ is the protected value within the unburned footprint area of fire *j*.
- *F* is a user-defined condition to warrant successful fire suppression along fuel breaks, which can be defined based on a single or multiple factors/parameters such as topography (e.g., slope), weather (e.g., temperature, wind), fire characteristics (e.g., fire size, flame length), treatment conditions (e.g., fuel type, time since last treatment, break’s width), suppression response time, etc. For example, in our test cases, a constant flame length threshold *FL* (also called the “escape flame length”) of either 2 feet, 4 feet or 8 feet was used as a parameter to define *F*; every fire with its maximum flame length along a fuel break exceeding the *FL* threshold will be assumed unstoppable by that break, regardless of whether the break is invested in or not.
- $C_{i}^{F}$ is the investment in the break *i* up to a level that can support effective fire suppression under the user-defined condition *F*.
- *B* is the total budget limit.
- ∅ is an empty set.
- $S_{j}^{F}$ is the set of fuel breaks that constitutes the ignition polygon of fire *j*. If the user-defined condition *F* is not met for fire *j*, *S*_*j*_ will be set to ∅. The process to derive the $S_{j}^{F}$ set is illustrated in Fig 1.

The objective function (1) maximizes the total landscape value protected by the FBN across all sample future wildfires. This value can be quantified as the total area avoided from burning, or other quantifiable values, such as timber volume or number of buildings within the avoided burning area etc. Eq (2) sets the upper limit for the total investment in fuel treatments within the FBN. Eq (3) is created only for the case when a fire ignition polygon exists for fire *j* (i.e., the fire is fully split by a set of fuel breaks in the FBN), and the condition *F* must be met for every fuel break segment constituting fire *j*’s ignition polygon (e.g., the maximum fire frame length along those fuel break segments does not exceed a predefined threshold). For example, if *x*_*i*_ = 1 for $\forall i\in S_{j}^{F}\neq \emptyset$, fire *j* will be contained within its ignition polygon (i.e., *y*_*j*_ = 1). Note that Eq (3) allows for *y*_*j*_ to receive the value of either 0 or 1, but due to impact of the objective function (1), the model has the incentive to force *y*_*j*_ to be 1.

The optimization model allows for searching the entire feasible decision solution space to find the best option for fuel break treatment. It can also be modified to examine predefined fuel break prioritization decisions. For example, considering a scenario with *S*′ being a set of fuel breaks in the FBN selected based on certain predetermined rules, two new equations can be added to capture the pre-determined fuel break selections represented by the *S*′ set:

$$x_{i}=1\;\forall i\in S^{\prime }$$ (4)

$$x_{i}=0\;\forall i\notin S^{\prime }$$ (5)

Eqs (4) and (5) reflect those rule-based decisions. Under this model set up, the other Eqs (1) to (3) would be used to calculate the objective function value based on the pre-selected decisions.

An example demonstrating the construction of a simple fuel break treatment problem is provided in S2 Appendix. In this example, a network with four fuel breaks and four simulated fire footprints are used to formulate an optimization model that select fuel breaks to minimize weighted sum of fire footprint areas protected from burning. Under the same assumptions, a rule-based model is also built for ranking and prioritizing fuel breaks selection. Solutions from these two modeling approaches are compared to emphasize the advantage of using optimization to achieve better outcomes.

## 3. Test cases

**Fig 2:**
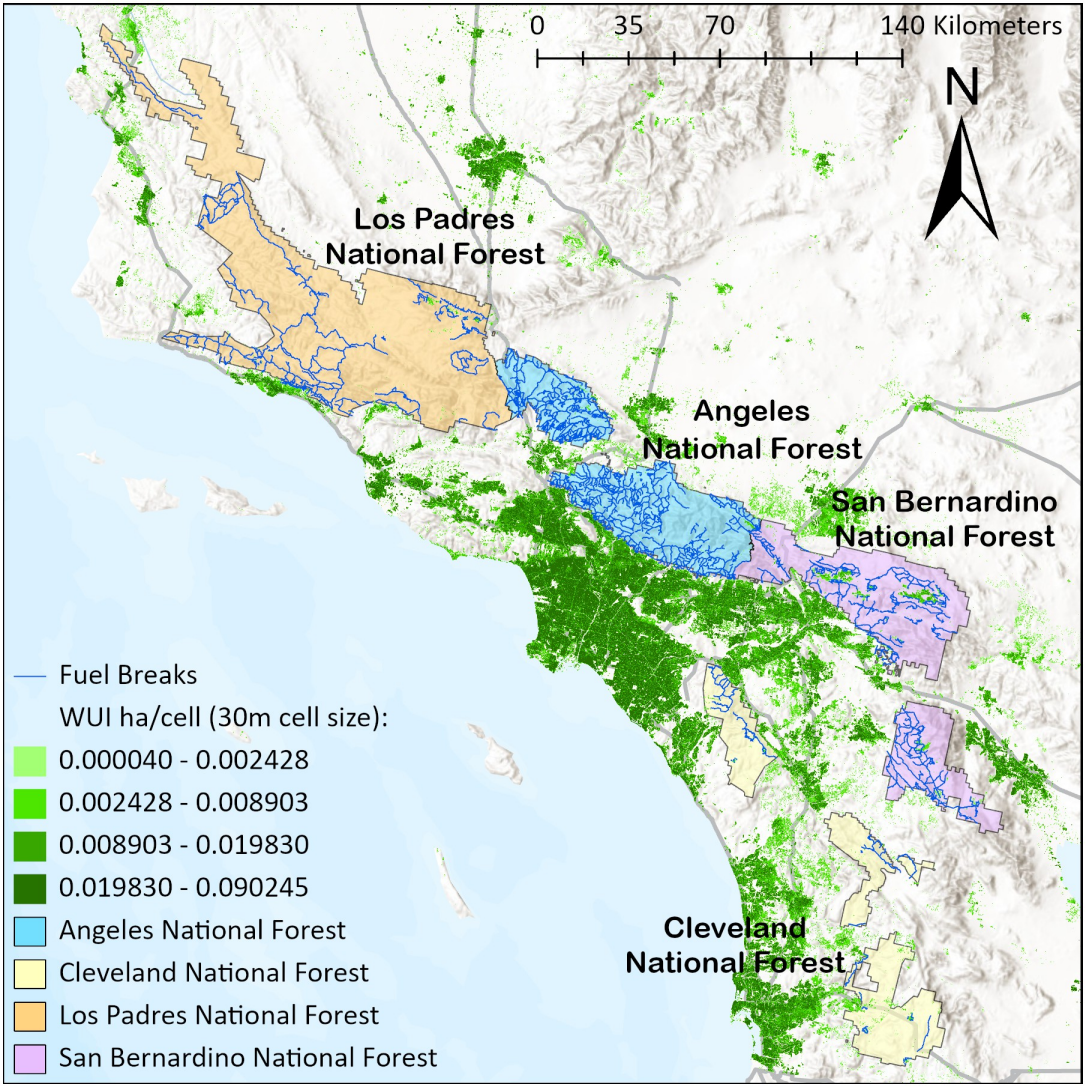
Southern California landscape. World Hillshade is used in the figure’s background (Sources: Esri, Airbus DS, USGS, NGA, NASA, CGIAR, N Robinson, NCEAS, NLS, OS, NMA, Geodatastyrelsen, Rijkswaterstaat, GSA, Geoland, FEMA, Intermap, and the GIS User Community).

Our study landscape encompasses four national forests, covering a total forest area of approximately 3.4 million hectares (Fig 2). It contains an existing FBN with 2,341 segments (lines vector [28]), spanning a total length of 5,021 kilometers. Anticipating future maintenance needs for this FBN to sustain its effectiveness, we explored how to prioritize fuel treatments within the FBN under a budget limit.

In the US, integrating wildfire simulation into landscape-scale fire risk assessment has become a common approach [52]. Fire-behavior tools, such as NEXUS, FVS-FFE, FARSITE, FLAMMAP, RANDIG, and FSIM, have been used for simulating wildfires [53]. FSIM, for instance, possesses the capability to simulate numerous wildfire events based on historical fire occurrences and weather, terrain and current fuel conditions within a landscape (https://www.firelab.org/project/fsim-wildfire-risk-simulation-software). Wildfires simulated by FSIM have been leveraged to support past FBN research, including simulation based fuel break prioritization [29], scenario-based optimization [27], and statistical analysis [28]. To facilitate our test cases, we used existing FSIM wildfire data generated and calibrated for the Southern California landscape by Pyrologix (https://pyrologix.com). FSIM was executed with 5000 replicates using the recent 15-year historical wildfire information in Southern California, including a mean annual burn probability of 0.0244, a mean annual number of 33 large fires, and a mean size of 5,141 acres for large fires. This extensive simulation process produced 259,654 fires, covering 6.9 million acres of land that extend beyond the 3.4 million acres of the four national forests within the landscape. Prior research has shown that 600 replicates could adequately represent the variance of fire situations across 10,000 fire seasons [54]. In our case, 5000 replications could effectively capture the historical fire size distribution in the study area. The resulting dataset includes fire footprints (polygons vector), their corresponding ignition locations (points vector) and flame lengths (raster with 270-meter cell size). In addition to the FSIM dataset, we also collected information on the landscape’s Wildland Urban Interface (WUI raster with 30-meter cell size, available at https://wildfirerisk.org/download), to facilitate testing different fire management objectives (Fig 2).

**Fig 3:**
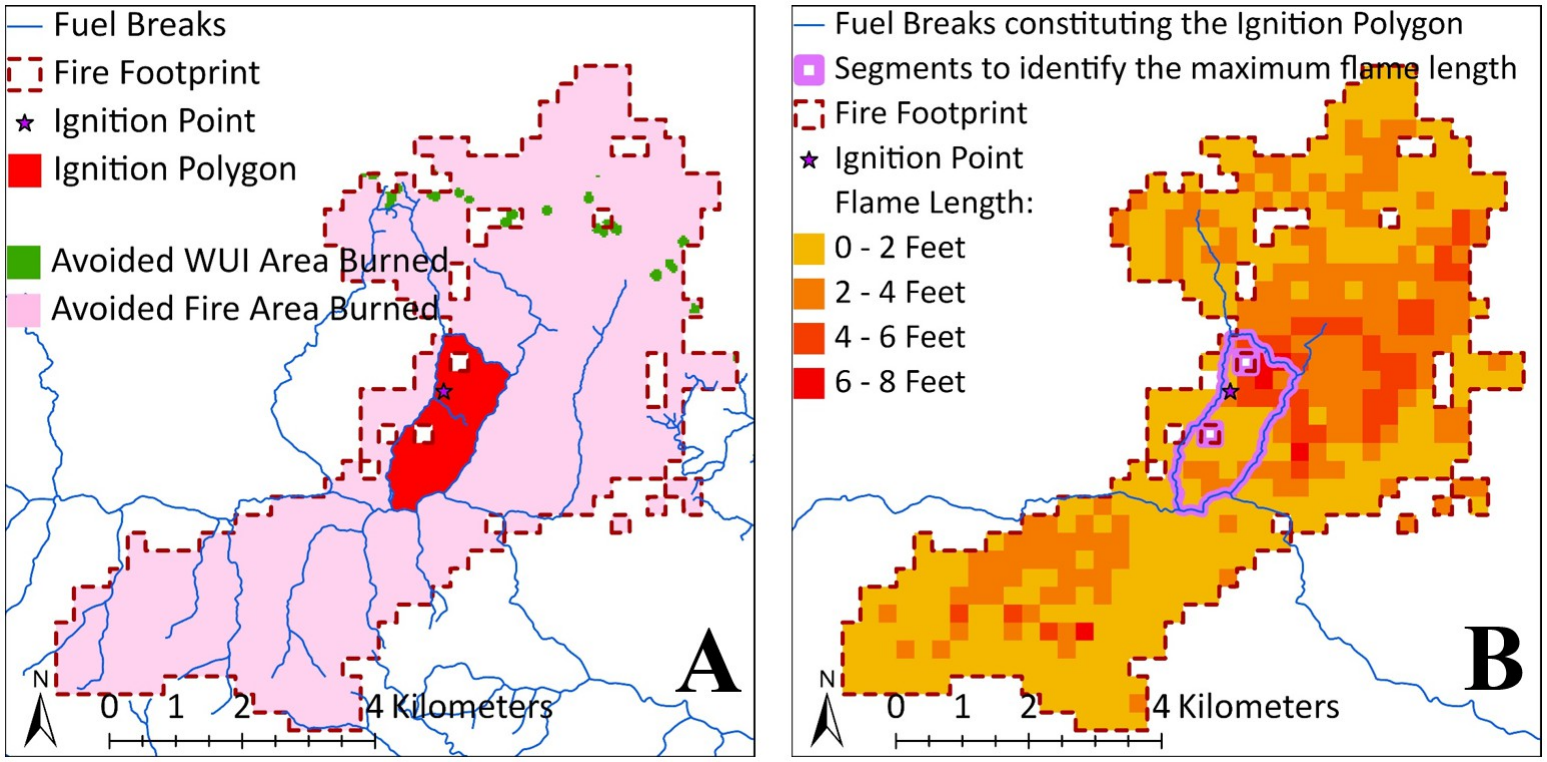
Illustration of data processing for 1 of 53,906 FSIM fires. **A**: Intersecting fuel breaks with the fire footprint to identify the ignition polygon and its associated fuel breaks. **B**: Intersecting the ignition polygon with the fire flame length raster to identify the highest flame length along the fuel break segments enclosing the ignition polygon.

Our data processing workflow consists of three main steps:

- Step 1: identifying fires for testing. We intersected all 259,654 fire footprints with the FBN and identified 205,748 footprints (79%) that have no interaction with the FBN (i.e., they do not encounter at least one fuel break). These 205,748 fires were excluded from all models since they have no impact on the model solutions. The remaining 53,906 fires (21%) were retained for model testing.
- Step 2: identifying parameters for the model constraints. For each of the 53,906 remaining fires, we followed a systematic process, as depicted in Fig 3. We first intersected each fire footprint and ignition point with the FBN to identify the ignition polygon (Fig 3A). Information of the fuel breaks defining the ignition polygon was extracted for use as model inputs (*M*_*j*_ and $S_{j}^{F}$). Next, we intersected the ignition polygon with the flame-length raster to identify raster cells overlapping the polygon’s boundary (i.e., cells alongside the purple fuel break segments in Fig 3B). From these cells, we selected the one with the highest flame length value to compare to a predefined flame length threshold (*FL*, where suppression is assumed to be effective). If this highest flame length value does not exceed *FL*, the fire is qualified for containment (note that fire is successfully contained only when all fuel breaks that constitute the ignition polygon are treated). In the other case when the highest flame length value exceeds *FL*, the fire is assumed to escape the ignition polygon regardless of treatment. Note that *FL* is just a simplification of the condition *F*, which can be customized for modeling more complex conditions to warrant successful fire suppression, as previously described in section **2.2**.
- Step 3: identifying parameters for the model objective function. For each fire (as illustrated in Fig 3), the footprint area outside of the ignition polygon (i.e., the pink areas in Fig 3A) represent the avoided fire area burned (**AFB**) assuming effective fire containment by the ignition polygon. The WUI map consists of raster cells, each covering 0.09 hectares, with raster values indicating the amount of WUI area (in hectares) within each cell. The WUI area may vary across cells, as shown by the gradient-green color in Fig 2. For each fire, we overlayed the AFB with the WUI map to identify which cells fall within the AFB (i.e., the green cells in Fig 3A), and sum their raster values to calculate the WUI area protected from burning, which is also referred to as the avoided WUI burned (**AWB**). We used both AFB and AWB to simplify the value at risk (*V*_*j*_) used in the test cases.

We designed scenarios to address several key challenges for wildfire management in Southern California, including WUI protection, suppression effectiveness in relation with fire intensity, and budget limitations. We systematically built and tested 120 optimization models, each represents a unique combination of the following scenario assumptions:

- Two policy preference scenarios that define the objective functions: maximizing either the total AFB or the total AWB across all fires.
- Two methods of selecting fire samples for modeling: “all fires”, and “no large fires” where 20% fires of the largest sizes were excluded. This exclusion helps focus our analysis on wildfires that have a better chance of being contained by fuel breaks. Prior research has shown that fuel break effectiveness declines under unfavorable conditions (e.g., strong wind, high temperature) that produce extreme fire behavior and unsafe working environments [55], which excludes potential suppression actions on many of the fuel breaks that interact with the largest wildfires.
- Three threshold values for the escape flame length (*FL*): 4 FT, 8 FT, and infinity (INF) were selected to represent increasingly challenging fire suppression scenarios. The 4 FT and 8 FT thresholds reflect realistic expectations for successful fire control: fires with flame lengths below 4 FT can typically be controlled using hand tools, while fires with flame lengths between 4 and 8 FT may require heavy equipment such as plows, dozers, pumpers, or retardant aircraft for effective suppression [56, 57]. The INF threshold was included to test an optimistic scenario that assumes fire suppression could be effective even under extreme fires. If the maximum flame length of a fire at the boundary of the ignition polygon exceeds the *FL* threshold, all fuel break segments along the polygon’s boundary will be considered ineffective in containing the fire regardless of whether they are treated or not. In this case, the fire is assumed to escape the ignition polygon and fully grow into its originally simulated footprint.
- Ten different budgets (*B*): 10–100% of the total FBN’s length. These percentages were selected to represent increasing levels of investment in treating the FBN, from minimal investment to full network coverage. Using a range of budget levels allows us to assess how different budget constraints impact fire management outcomes. We used simplified assumptions that the treatment cost for each break ($C_{i}^{F}$) is equal to the length of that break, and the total length of the selected breaks for treatment could not exceed a predefined budget limit *B*. To implement this assumption, Eq (2) is replaced by Eq (6):
$$\sum_{i}L_{i}x_{i}\leq B$$ (6)
  Where *L*_*i*_ is the length of fuel break *i*, and *B* is the maximum total length of fuel breaks that can be invested in (e.g., 10%, 20%, 30% of the network length).

In this study, we also designed a test case to evaluate the advantage of using the optimization (**OPT**) approach over a rule-based selection (**RBS**) approach under a set of predefined assumptions. For the RBS approach, we assumed that all fuel breaks in the FBN were treated to calculate the cost effectiveness of each individual break (*E*_*i*_) by Eq (7). Fuel breaks are then selected into the *S*′ set based on *E*_*i*_, from higher to lower values of *E*_*i*_ until the budget *B* is reached (equal *B*, or close to but not exceeding *B*). The *S*′ set was used in Eqs (4) and (5) to define the predetermined fuel break selections.

$$E_{i}=\sum_{j}\frac{V_{j}}{C_{i}^{F}}\;\forall i$$ (7)

Where *j* belongs to the set of fires that are either contained by break *i* individually or by the collaboration of break *i* with other breaks in the FBN, assuming that all breaks in the FBN are treated.

For each of the 120 OPT models, we built a corresponding RBS model as described above. By comparing solutions between the OPT and RBS models, we can evaluate the benefits of using the optimization approach.

## 4. Results

### 4.1. The optimization model solutions

All models were solved by using IBM’s ILOG-CPLEX 12.8 on a 64-bit workstation equipped with a dual-core 2.5 GHZ processor and 32 GB of RAM. The entire process of running 120 OPT models took approximately 17 hours (up to 1.8 hours to complete an individual run as shown in S3.1 Fig in S3 Appendix), while it took only 0.3 hours to complete all 120 corresponding RBS models. Here, the total run time was calculated as the sum of time to read and process model inputs, time to solve models in CPLEX, and time to write model outputs.

**Fig 4:**
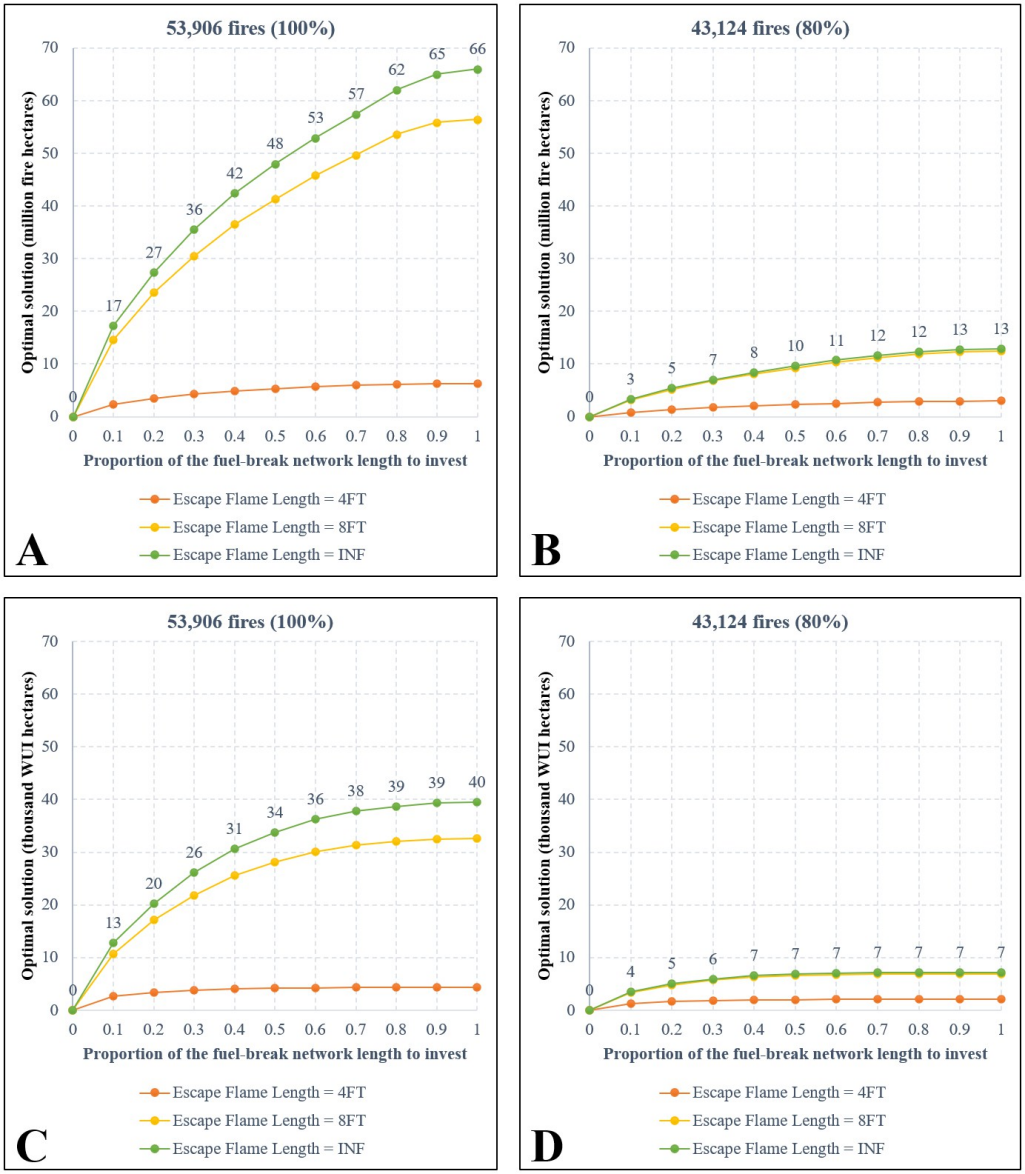
Objective function values of the optimal solutions. **A**: Maximizing AFB, all fires. **B**: Maximizing AFB, no large fires. **C**: Maximizing AWB, all fires. **D**: Maximizing AWB, no large fires. Note that the unit is “million fire acres” in **A** and **C**, while it is “thousand WUI acres” in **B** and **D**. Numbers are shown for the green lines only.

Fig 4 shows the optimal objective values from all the optimization models that are organized into four categories (**A, B, C, D**) based on the type of objective functions and the number of modeled fires. Both assumptions of the objective functions including “maximizing AFB” (Fig 4A and 4B) and “maximizing AWB” (Fig 4C and 4D) show an obvious “diminishing marginal returns” effect as increasing the budget limit (i.e., the proportion of the FBN to invest by length) will result in smaller increases in the optimal objective values. For example, Fig 4 shows that increasing the budget from 0 to 0.5 would prevent 119 million acres from burning by fires, while increasing the budget from 0.5 to 1 would additionally protect an extra 44 million acres (note that overlapped protected acres were included in these calculations). In all cases, the highest returns were obtained when the budget was increased from 0 to 0.1 of the total FBN’s length. More investment exceeding a certain level may result in significantly low marginal returns. For instance, Fig 4D shows no benefit when investing in more than 70% of the total FBN’s length. The greatest benefit comes from containing large fires, as illustrated by significantly flatter lines in Fig 4B and 4D (where the top 20% largest fires are excluded from modeling), in comparison to Fig 4A and 4C.

Each of the four panels (**A, B, C, D**) in Fig 4 presents the optimal solutions under a specific assumption of the escaping fire flame length threshold. If investment can assure successful suppression of fires with flame lengths not exceeding 8 feet, then the effectiveness of the solutions is comparable to assuming successful suppression of fires with any (INF) flame lengths, especially when large fires are not considered in the models. This is evident from the nearly overlapping yellow and green lines in Fig 4B and 4D. However, assuming a 4-foot escape flame length significantly reduces the effectiveness of the solutions compared to the other two assumptions (8FT and INF).

**Fig 5:**
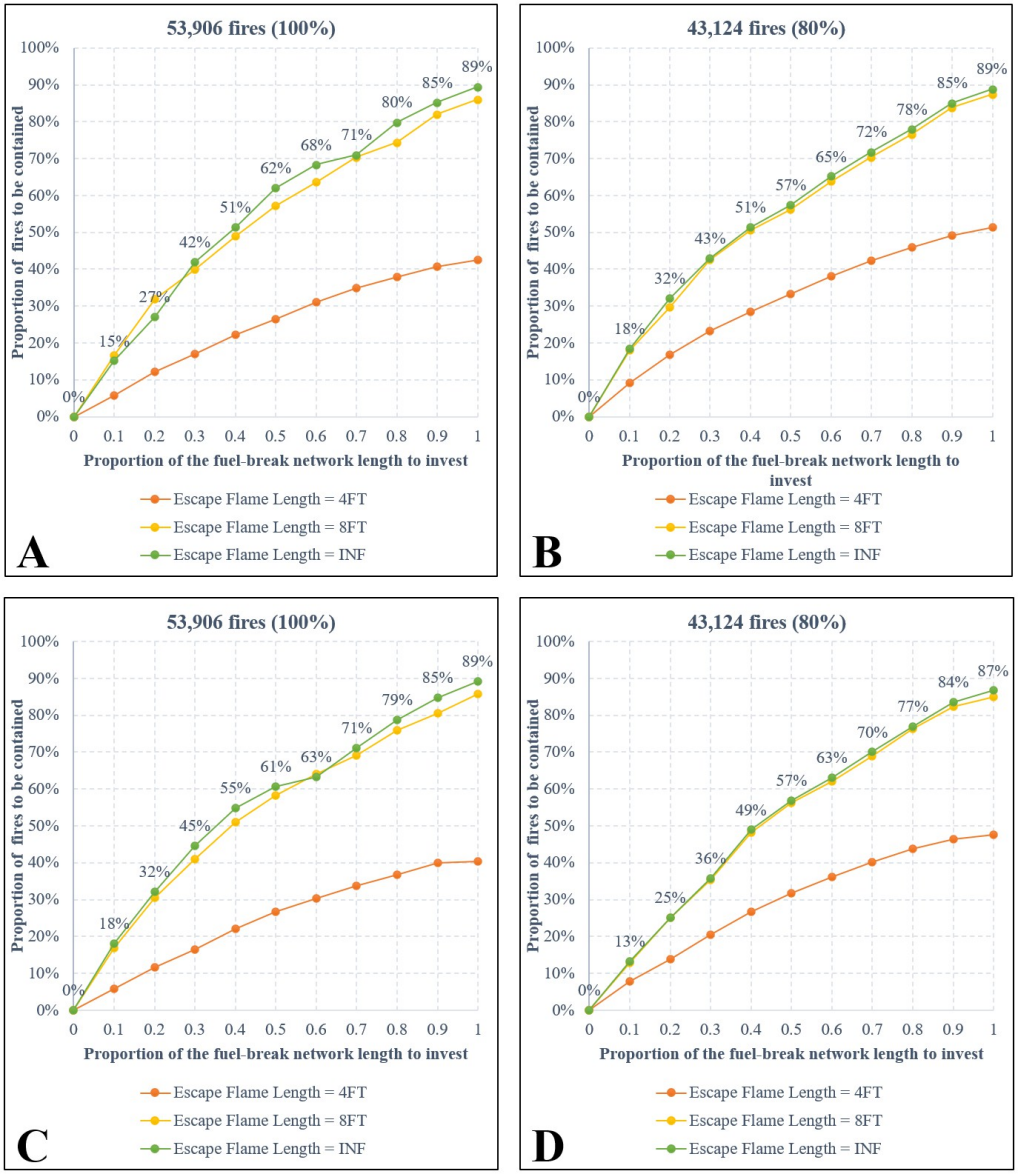
Proportion (percentage) of number of fires being contained in the optimal solutions. **A**: Maximizing AFB, all fires. **B**: Maximizing AFB, no large fires. **C**: Maximizing AWB, all fires. **D**: Maximizing AWB, no large fires. Note that in **A** and **C**, the percentage is calculated by the number of contained fires divided by 53,906; while in **B** and **D**, the percentage is calculated by the number of contained fires divided by 43,124. Percentages are shown for the green lines only.

Fig 5 shows a very large number of fires (87–89% of the total tested fires) being contained under the assumptions that the entire FBN is treated and fires with any flame length (INF) could not breach the treated breaks. Note that these percentages were calculated based on only 53,906 fires (or 43,121 when excluding large fires) that encountered (touched) at least one fuel break. The findings suggest that the spatial arrangement of the breaks in the network is well-designed for a portion of the Southern California landscape, providing a high likelihood of successfully containing the fires that encounter treated fuel breaks. Across test cases, increasing the treatment budget (i.e., treating a greater proportion of the total FBN length) resulted in a larger number of fires being contained (Fig 5), but the objective function value may not improve proportionally. For example, when assuming a 4-foot flame length suppression effectiveness, doubling treatment from 50% to 100% of the network length significantly increased the number of contained fires from 27,771 to 48,223 (**AFB** objective, green line in Fig 5A) or from 32,659 to 48.088 (**AFB** objective, green line in Fig 5C). However, improvement in the **AWB** objective value was less substantial (from 34 to 40, Fig 4C) compared to the **AFB** objective (from 48 to 66, Fig 4A).

**Fig 6:**
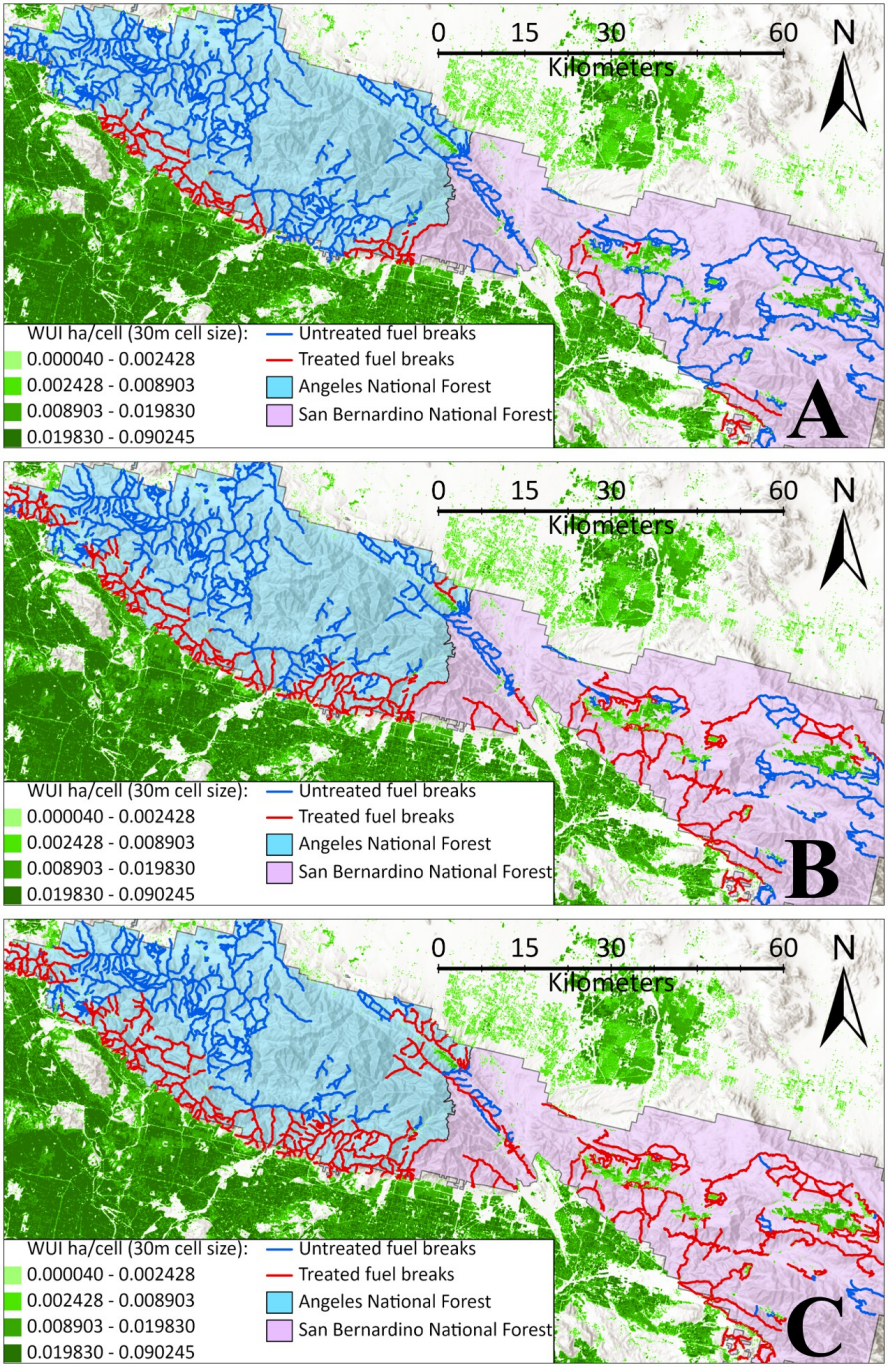
Some optimization treatment layouts considering the objective function of maximizing AWB and 20% largest fires are excluded. **A**: Treating 10% FBN length. **B**: Treating 30% FBN length. **C**: Treating 50% FBN length. This figure only shows a portion of the Southern California landscape. More detailed solutions are presented in S3.4, S3.5 Figs in S3 Appendix. World Hillshade is used in the figure’s background (Sources: Esri, Airbus DS, USGS, NGA, NASA, CGIAR, N Robinson, NCEAS, NLS, OS, NMA, Geodatastyrelsen, Rijkswaterstaat, GSA, Geoland, FEMA, Intermap, and the GIS User Community).

The spatial layouts of selected breaks in the optimal model solutions can be examined in an interactive Google Earth Engine web-application, available at https://thumit.users.earthengine.app/view/landscape-fuelbreak-prioritization. Out of the 120 OPT models, we selected 60 solution layouts for illustration in S3.2, S3.3, S3.4, S3.5 Figs in S3 Appendix. These layouts represent 60 model cases with budget limits of 0.1, 0.3, 0.5, 0.7, and 0.9. One notable distinction between the layouts is that when the objective function changes from “maximizing AFB” to “maximizing AWB”, the selected breaks tend to be allocated closer to the forest boundary, especially when large fires are excluded from the models (Fig 6 and S3.4, S3.5 Figs in S3 Appendix for more details). When the objective is to maximize AFB, the model prioritizes placing fuel breaks in locations where fires can be effectively contained before spreading over larger areas, resulting in a more dispersed placement of fuel breaks across the landscape. In contrast, when the objective is to maximize AWB, fuel breaks are allocated closer to areas with a higher concentration of WUI assets, even if this leads to less overall fire containment. This shift in fuel break layout occurs because WUI areas are primarily situated outside of the forests (Figs 2 and 6), investing in breaks near the forest boundary will have a more significant impact on WUI protection. Another finding is that when the budget is limited, the optimal solutions prioritize investing in fuel breaks within the two forests at the bottom half of the landscape (i.e., San Bernardino and Cleveland), after which investments expand to the other two forests at the top half of the landscape (i.e., Los Padres and Angeles). We suppose that this is due to the simulated fires having more interaction with San Bernardino and Cleveland forests than the other two forests. Interestingly in many cases, increasing the budget while keeping all other model assumptions fixed would generally result in expanding rather than drastically changing the breaks selected in the optimal break layouts. While this pattern may be landscape-specific, it can be useful for planning continuous investments when budget is a limiting factor. Lastly, the models assuming 8FT and INF escape flame length produced nearly identical solution layouts (S3.2 S3.3, S3.4, S3.5 Figs, in S3 Appendix). This can be explained by the small difference between these two assumptions regarding the number of fires would escape, as illustrated by Fig 5.

### 4.2. Comparing solutions from the optimization models and the rule-based selection models

**Fig 7:**
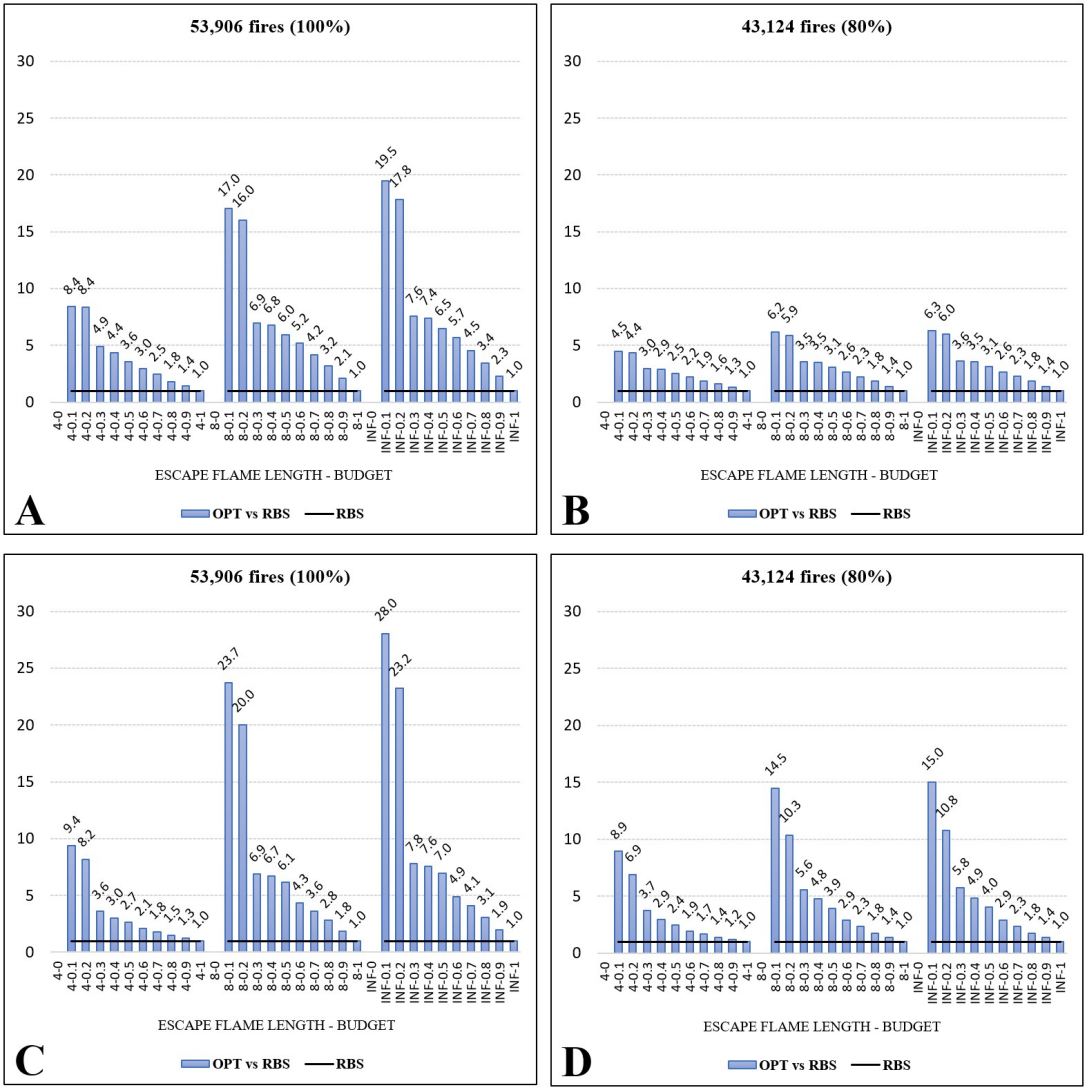
Comparing solutions from the optimization (OPT) models and the rule-based selection (RBS) models. **A**: Maximizing AFB, all fires. **B**: Maximizing AFB, no large fires. **C**: Maximizing AWB, all fires. **D**: Maximizing AWB, no large fires. Note that solutions presented here are rescaled (divided by the objective function values from the RBS models). The original solutions can be found in Table S3.1 in S3 Appendix.

The OPT models produced substantially better solutions in comparation to the corresponding RBS models (i.e., greater objective function values, as shown in Fig 7 and Table S3.1 in S3 Appendix). In the best-case scenarios, typically under low budgets, the OPT model objective function values can be 28 times better than those from the RBS model solutions (Fig 7C). The superiority of the OPT models over the RBS models decreases when larger fires are excluded from testing (Fig 7B and 7D).

Compared to the OPT models, RBS models are less efficient because they lack the capability to account for the dynamic coordination of limited fuel breaks selected for fuel treatments. According to the RBS approach, all fuel breaks in the FBN are assumed treated to calculate the effectiveness score for each individual fuel break (*E*_*i*_); under a limited treatment budget, only a smaller subset of fuel breaks with the highest *E*_*i*_ scores will be selected for investment. However, selecting the highest-ranking fuel breaks cannot guarantee that they would be the best selections for fire containment. For example, some breaks may have high ranking scores due to their coordination with many other low-ranking breaks to stop multiple fires, but selecting only those high-ranking breaks without selecting their corresponding low-ranking ones will result in less effective fire containment. While RBS models do not allow for changing the values of decision variables (*x*_*i*_) predetermined by the ranking rule, the OPT approach is more flexible in allowing all decision variables *x*_*i*_ to change to find the best coordination of fuel breaks for fire containment. Our findings that the optimization approach (OPT models) produced significantly better results compared to the rule-based selection approach (RBS models) emphasize the importance of considering the spatial interaction among fuel treatments within an FBN to make more effective and efficient selection decisions.

## 5. Discussion

Research exploring the potential benefits of FBNs in wildfire management and planning can provide insights into the important role of fuel breaks. In this study, we developed an optimization model to examine the utility of an FBN to maximize the total landscape value to be protected (avoided area burned or avoided WUI area burned) from potential future wildfires. Our model addresses the challenge of prioritizing fuel treatments within an FBN under a limited treatment budget. It can assist wildfire managers in developing alternative plans for fuel treatment prioritization and evaluating their tradeoffs to inform strategic FBN planning decisions.

Few optimization studies have been implemented to look at tradeoffs in FBN management by considering its interactions with potential fire behavior and fire suppression opportunities. Our model is built on the assumption that linear fuel breaks are designed to support safer and more effective fire suppression. Thus, it explicitly incorporates fire behavior and fire suppression effectiveness by assuming a fire may be effectively contained when it is enclosed by a set of fuel breaks; the model only considers these breaks effective if two conditions are met: 1) those fuel breaks are treated to allow for suppression operations, and 2) suppression along those treated fuel breaks would be successful when fire intensity does not exceed a predefined threshold. Explicitly considering the coordination of treated fuel breaks to facilitate fire suppression and containment represents a unique modeling approach that distinguishes our research from existing FBN modeling studies.

Our optimization model is designed with a compact MIP formulation that can be customized to adapt to solving various fuel break prioritization problems in large and diverse landscapes. Integrating additional data can expand the model capability to consider other management objectives and analyze their tradeoffs. For instance, in the Great Basin region of the United States where there is a growing need to protect native species [58], a model objective focused on maximizing habitat protection might be more appropriate than WUI protection or fire size reduction. Data on the landscape’s ecological values can also be incorporated to build additional model constraints that examine the ecological impacts of fuel breaks in a landscape. These impacts, such as habitat fragmentation, have been addressed by empirical and simulation-based FBN studies [59–61], but have rarely been explored using optimization models. Including such ecological considerations to best meet management goals can improve the model’s applicability. However, it’s important to balance the inclusion of these factors against computational performance to ensure that the model remains solvable to deliver optimal outcomes.

The capability to solve landscape scale (i.e., large scale) problems is another notable aspect of our model. Existing optimization models in fuel treatment management often face the challenge of solving landscape-scale problems due to computational issues [35, 62, 63]. Consequently, heuristics and simulation-optimization have been utilized in those models to allow for solving larger-size problems, but they can only guarantee solution feasibility instead of optimality [64–66]. Our test cases demonstrated that the optimization model can consistently provide superior solutions compared to a rule-based heuristic selection approach. The optimization model also performs well, evidenced by the solution time which took less than two hours for solving a regional-scale fuel break planning problem (see Fig S3.1 in S3 Appendix). In optimization problems, particularly complex ones, it is common to stop when a near optimal solution is found (e.g., within a 5% gap from the tur optimum). In our test cases, all models achieved true optimal solutions (with 0% gap), which demonstrates the computational efficiency of our proposed optimization approach in producing quality results. It is important to acknowledge that model performance was evaluated based on the two methods (optimization vs rule-based heuristic selection) with specific designs. Comparing the efficiency of our optimization model with other established methods would be interesting, but this is a broad topic beyond the scope of our study.

### 5.1. Insights for regional fuel break planning

In 2020, the US Bureau of Land Management released a plan to fund the construction and maintenance of up to 11,000 miles (17,700 km) of strategic fuel breaks across several western states where wildfire threats are critical, including Idaho, Oregon, Washington, California, Nevada and Utah (https://www.blm.gov/press-release/blm-releases-final-plan-construct-and-maintain-11000-miles-fuel-breaks-great-basin). With continuous efforts to construct and maintain extensive fuel break networks in these states, insights into fuel break prioritization can benefit fire management decision-making, especially in situations with financial constraints.

We tested the MIP model on a case study prioritizing fuel break treatment in a Southern California landscape. We constructed multiple test cases to explore the optimal model solutions (i.e., how fuel breaks are selected for fuel treatment maintenance) in response to fire management scenarios that varied the following characteristics: different management priorities (i.e., WUI protection or fire-size reduction), including or excluding large fires in the models, different flame length thresholds beyond which fires are considered uncontainable, and a range of budget limits for investment in treating the FBN. FSIM-simulated wildfires were collected and used to facilitate model testing.

Several important insights can be derived from testing our optimization model on the Southern California landscape. Overall, our findings suggest that a higher investment level in the FBN would protect more of the landscape’s values from possible future wildfires. The marginal return of investment is higher at a lower budget level, and diminishes when the budget level increases, especially when the management priority is to protect the WUI and/or when large fires are excluded from fire management consideration. Investments beyond a certain level may yield no substantial changes in protecting the landscape’s values (such as when prioritizing WUI protection against smaller fires as illustrated in Fig 4D). Our tests also suggest that the spatial locations of selected fuel breaks would be driven by different management priorities. For example, more breaks will be selected near WUI areas when protecting WUI is the main objective. When increasing the budget level, the spatial fuel break layout from the higher budget is generally an expansion of the layout from the lower budget; thus, fire managers could consider making small initial investments in treating fuel breaks when budget is limited, and expanding the scope of treatments when additional funds become available. It is also worth noting that 79% of all the simulated wildfires have no interaction with the FBN and were excluded from the models because they do not engage with fuel breaks. Expanding the existing FBN to allow for more landscape protection can be considered but should be carefully planned, as constructing new fuel breaks (such as into the WUI areas) may prompt negative responses [26]. While we are aware of the 2022 National Strategy that supports more fuel reduction across the US, we did not consider any plans to construct new fuel breaks in the Southern California landscape since it is beyond the scope of our analysis.

### 5.2. Applications, limitations, and potential improvements

Having a candidate FBN preidentified is necessary for the application of our model. The model can be used to evaluate the return on investment for those candidate fuel breaks and suggest the optimal investment options to achieve the prioritized fire management objectives. This assumption aligns with the real-world planning situation in Southern California, where maintaining a well-established FBN requires regular prioritization of fuel break treatments. In landscapes that do not have candidate FBN layouts in place, preliminary spatial analyses or planning would be necessary to identify the candidate fuel breaks before implementing our prioritization model. An inherent limitation of our model is that solution quality will strongly depend on the preliminary identification of the plausible fuel breaks, which is a typical challenge in FBN modeling. Unlike area-wide fuel treatments which can cover a landscape entirely by a limited set of treatment units, linear fuel breaks can be constructed with diverse shapes and lengths in a landscape, resulting in unlimited options for fuel break configuration. The preliminary identification of fuel break candidates establishes a finite solution space, allowing for evaluation by the optimization model.

While our study exclusively focuses on FBN optimization modeling, it is important to acknowledge numerous optimization models that have been developed for area-wide fuel treatments. Instead of treating linear fuel breaks, these models consider treatment units represented by either raster cells [46, 67–71], nodes [72, 73], or polygons [45, 47, 74]. Besides the difference in delineating treatment units, linear fuel breaks and area-wide fuel treatment are also different in their primary use. Area-wide fuel treatments focus more on modifying fire behavior or effects as fire moves across the treated areas, while linear fuel breaks primarily aim to facilitate wildfire suppression along contiguous and narrow land strips [24, 25]. A strategic wildfire management plan that incorporates both approaches could address criticisms directed at solely using fuel breaks [26]. Integrating both fuel breaks and area-wide fuel treatments into an optimization model is a potential future direction for our research, as to our knowledge, this has not been addressed by existing studies.

It is worth noting that we have constructed test cases based on some specific assumptions including the land value to be protected (*V*_*j*_), the cost associated with investing in each break ($C_{i}^{F}$), and suppression response efficiency under specific fire intensity thresholds (*FL*). To apply our model in a more practical setting, it may be necessary to incorporate greater model details and more accurate model parameters. For instance, the protected value should account for a range of economic, environmental and social factors, including buildings, timber, wildlife, and recreational value, among others. Similarly, the cost for treating a break should take into account its current condition, such as the break type, break width, topography and time since last treatment at each break’s location. Additionally, suppression details such as cost and response time, could be explicitly integrated into the model for a more realistic evaluation of suppression effectiveness [75]. The model objective can also be modified to maximize the net value, considering benefits from protected land areas, losses due to burning, and costs from both fuel treatment and suppression activities (see S4 Appendix). While incorporating these additional details is a worthy goal, it also presents challenges due to increased model complexity and data requirements [68, 69, 75, 76].

Our assumption of effective fire containment alongside the “ignition polygons” can hold true when fire crews have enough time to respond, which may not be possible if the polygon size is too small and the fire spreads too quickly for timely reaction. In such cases, fire can cross the breaks before any suppression efforts occur. A possible enhancement to our model is using fuel breaks beyond the ignition polygon to form additional contingent containment areas. This requires extensive data processing to identify multiple containable polygons and also requires modifications to the model formulation to evaluate various containment outcomes.

Another limitation of our model is that it does not capture the impact of partial containment on fire size reduction. Even when treated fuel breaks do not fully enclose a fire (i.e., partial containment), they can still be effective in blocking or slowing fire spread in certain directions, and therefore the final fire size may decrease compared to the originally simulated fire size. Because our model does not account for the partial containment effect, it may produce solutions that favor full containment of fewer fires rather than partial containment of a larger number of fires, which could be an important consideration when prioritizing WUI protection. Incorporating the impact of partial containment into the model is theoretically feasible by rerunning FSIM simulations for every fire to pre-estimate fire size reductions associated with different fuel break treatment scenarios. However, this is impractical because it would require an enormous amount of time and resources to rerun FSIM. Developing a practical method to account for partial containment is an area of research that we aim to explore in future studies.

It would be also beneficial to improve the modeling approach to account for uncertainties from both management and disturbances, as they may affect the reliability of predicted outcomes [77]. For example, suppression response time and safety can be affected by unexpected wind conditions that cause intense crowning and long-distance spotting fires [78, 79]. By further incorporating these details, we can improve the accuracy and effectiveness of our optimization approach, making it more useful in practical applications. These also represent potential future research.

## 6. Conclusions

This paper introduces an optimization modeling approach aimed at maximizing landscape protection against wildfires. The primary objective is prioritizing fuel treatments within a fuel break network to facilitate rapid wildfire suppression. Beyond developing a theoretically sound modeling method, we strongly focused on ensuring the model scalability and adaptability, which can promote its flexible and practical applications. We built 120 scenarios to demonstrate a potential application of our optimization model for prioritizing fuel treatments within a large fuel break network in Southern California, under varying treatment budgets and other fire management assumptions. This model can adapt to solving the fuel break planning problems in diverse landscapes. Its capability to inform strategic fuel break treatment decisions can draw interest from wildfire researchers and managers.

## Supporting information

S1 Appendix Ignition polygon approach. (DOCX)

S2 Appendix An example of solving a fuel break prioritization problem using the optimization approach and the rule-based selection approach. (DOCX)

S3 Appendix Optimization model solutions. (DOCX)

S4 Appendix Modification of the optimization model to maximize the net value. (DOCX)
