# Supplementary material for: An optimization model to prioritize fuel treatments within a landscape fuel break network
Source: PLoS One. 2024 Dec 17;19(12):e0313591. doi: 10.1371/journal.pone.0313591 (PMC11651606; doi:10.1371/journal.pone.0313591)
Supplement: S1 Appendix — (DOCX) [file pone.0313591.s001.docx]

**S1 Appendix. Ignition polygon approach**

The simulation based approach by Aparício et al. (2022) intersects each simulated fire footprint with the fuel break network to identify whether the fire can be contained. When a fire can be fully split by a set of fuel breaks into multiple polygons, the smallest polygon surrounding the fire’s ignition point (i.e., the “ignition polygon”, as illustrated in **Fig S1.1**) is identified. The portion of the fire footprint within the “ignition polygon” is assumed to be burned and contained, while the remaining portion of the fire footprint is considered protected. Across all fires, the weighted sum of footprint areas protected from burning (e.g., the black areas in **Fig S1**) is calculated to compute the effectiveness of each individual fuel break (**Table S1.1**). Subsequently, fuel breaks can be prioritized based on their effectiveness ranking.


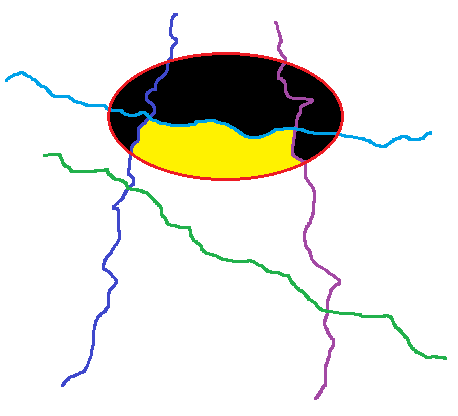

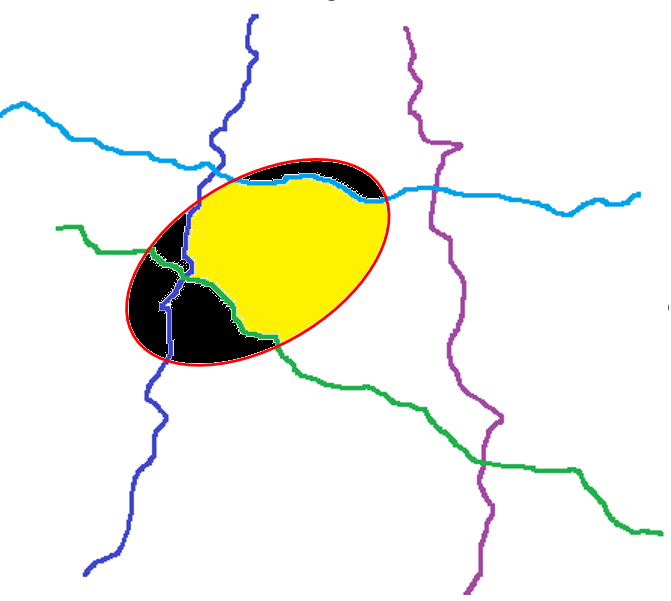

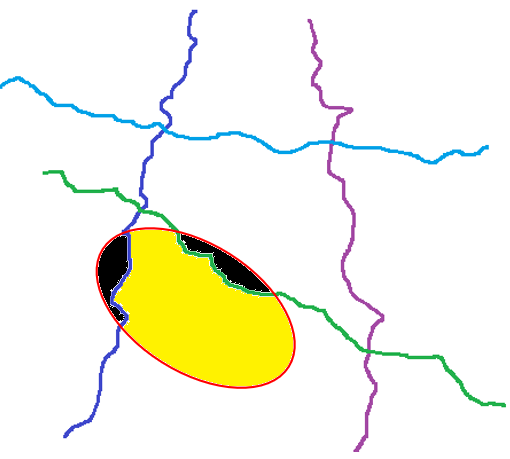

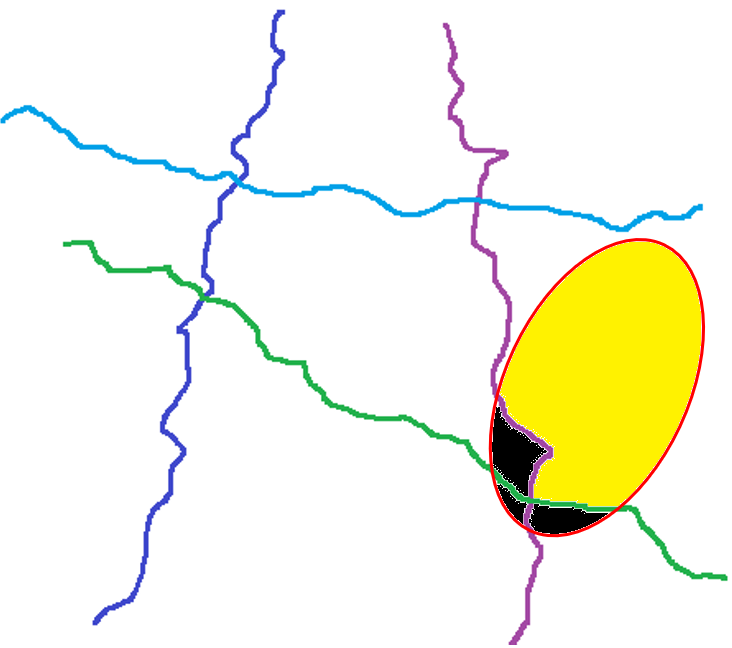


Fuel Break 1

Fuel Break 2

Fuel Break 3

Fuel Break 4

Fuel Break 1

Fuel Break 2

Fuel Break 3

Fuel Break 4

Fuel Break 1

Fuel Break 2

Fuel Break 3

Fuel Break 4

Fuel Break 1

Fuel Break 2

Fuel Break 3

Fuel Break 4

**A**

**B**

**C**

**D**

Ignition Polygon

Ignition Polygon

Ignition Polygon

Ignition Polygon

**Fig S1.1.** An example to illustrate the method of intersecting a fuel break network with simulated fire footprints to identify fire containment. In this example, a simple network with four fuel breaks (assumed equal lengths) and four simulated fire footprints (assumed elliptical shapes) are used. The “ignition polygons” (yellow) represents portions of the fire footprints that are contained by fuel breaks, while the black areas represent the remaining footprint portions that are avoided from burning.

**Table S1.1.** An example to demonstrate the approach used by Aparício et al. (2022) for ranking and prioritizing fuel breaks based on their effectiveness in fire containment. For this example, the fuel breaks and the simulated fires depicted in **Fig S1.1** are used.

| Fire ID | Fuel breaks that form the ignition polygon (FIP) | Avoided fire area burned (AFB) | Effectiveness of an individual fuel break calculated for each fire, based on AFB weighted by the length of intersection between the fire footprint and each fuel break segment within the FIP | | | |
| --- | --- | --- | --- | --- | --- | --- |
|  |  |  | Fuel break 1 | Fuel break 2 | Fuel break 3 | Fuel break 4 |
| 1 (Fig. 1A) | 1, 2, 4 | 600 | 120 | 360 |  | 120 |
| 2 (Fig. 1B) | 1, 2, 3 | 300 | 60 | 120 | 120 |  |
| 3 (Fig. 1C) | 1, 3 | 120 | 60 |  | 60 |  |
| 4 (Fig. 1D) | 3,4 | 80 |  |  | 40 | 40 |
| Fuel break effectiveness across all fires | | | 240 | 480 | 220 | 160 |
| Fuel break ranking by total effectiveness | | | 2nd | 1st | 3rd | 4rd |

**Reference**

Aparício, B. A., Alcasena, F., Ager, A., Chung, W., Pereira, J. M., & Sá, A. C. (2022). Evaluating priority locations and potential benefits for building a nation-wide fuel break network in Portugal. *Journal of Environmental Management*, 320, 115920.
