# Supplementary material for: An optimization model to prioritize fuel treatments within a landscape fuel break network
Source: PLoS One. 2024 Dec 17;19(12):e0313591. doi: 10.1371/journal.pone.0313591 (PMC11651606; doi:10.1371/journal.pone.0313591)
Supplement: S2 Appendix — (DOCX) [file pone.0313591.s002.docx]

**S2 Appendix. An example of solving a fuel break prioritization problem using the optimization approach and the rule-based selection approach**

To demonstrate how the formulation works, we use the example fuel break network (FBN) and simulated fires as illustrated in **Fig S1.1** and **Table S1.1**. To set up a complete problem, we assume that the budget would only allow for treating no more than two fuel breaks in the FBN ($B=2$), and the cost for treating each fuel break would be a constant value of one ($C_{i}^{F}=1$). We also assume that the condition *F* is met for every fire (i.e., all fires are non-intense such that suppression will be always successful along the treated fuel breaks).

Given these assumptions, the optimization model, including equations (1) to (3) in the main manuscript (also reprinted here), can be fully expressed by equations (S1) to (S6):

*Maximize:*

$\sum_{j} V_{j}y_{j}$ (1): $600y_{1}+300y_{2}+120y_{3}+80y_{4}$ (S1)

*Subject to:*

$\sum_{i} C_{i}^{F}x_{i}\leq B$ (2): $x_{1}+x_{2}+x_{3}+x_{4}\leq2$ (S2)

$y_{j}\leq\frac{1}{M_{j}}\sum_{i\in S_{j}^{F}\neq\emptyset} x_{i}\forall j$ (3): $y_{1}\leq\frac{1}{3}\left( x_{1}+x_{2}+x_{4} \right)$ (S3)

$y_{2}\leq\frac{1}{3}\left( x_{1}+x_{2}+x_{3} \right)$ (S4)

$y_{3}\leq\frac{1}{2}\left( x_{1}+x_{3} \right)$ (S5)

$y_{4}\leq\frac{1}{2}\left( x_{3}+x_{4} \right)$ (S6)

The model solution would be selecting fuel breaks 1 and 3 for treatments ($x_{1},x_{3}=1$ and $x_{2}, x_{4}=0$), which result in the optimal objective function value of 120 ($y_{3}=1$ and ${y_{1},y}_{2},y_{4}=0$).

Under the same assumptions, a ruled-based approach can be used to define a fuel break prioritization scenario. For example, the rule-based approach proposed by Aparício et al. (2022) would select fuel breaks 2 and 1 because they have the highest ranks (see **Table 1** in the main manuscript). The consequence of these selections can be examined by using equations (1) to (5) in the main manuscript, which can be fully expressed by equations (S1) to (S10). Under this selection scenario, none of the four simulated fires (**Fig S1.1**) can be effectively contained by the ignition polygons (${y_{1},y}_{2},y_{3},y_{4}=0$), resulting in an objective function value of zero. Note that fire containment opportunities beyond the ignition polygons are not taken into account.

$x_{1}=1$ (S7)

$x_{2}=1$ (S8)

$x_{3}=0$ (S9)

$x_{4}=0$ (S10)

The computational complexity of the optimization (OPT) model is substantially higher than that of the ruled-based selection (RBS) model due to the exhaustive evaluation of feasible solutions. In the OPT model, each fuel break has two decision variables: to treat or not to treat. Given that our study landscape contains 2,341 fuel breaks, the total number of feasible solutions is $2^{2341}$, which creates a massive solution space that requires extensive computational time (hours) to find the optimal solution. In contrast, the RBS model utilizes a preprocessing step to rank fuel breaks (before running the model), allowing it to pre-determine a subset of breaks to treat based on these rankings. Consequently, the RBS model does not need to evaluate all possible treatment combinations; it simply coordinates the selected fuel breaks to assess outcomes, enabling it to solve in a matter of seconds.

Improving the computational efficiency of the OPT model poses significant challenges. Our team has invested considerable time in testing various formulations to identify the one that provides the best performance. Future efforts could focus on decomposition approaches or other relaxation techniques to enhance computational efficiency while maintaining the model's integrity and accuracy.

**Reference**

Aparício, B. A., Alcasena, F., Ager, A., Chung, W., Pereira, J. M., & Sá, A. C. (2022). Evaluating priority locations and potential benefits for building a nation-wide fuel break network in Portugal. *Journal of Environmental Management*, 320, 115920.
