# Supplementary material for: An optimization model to prioritize fuel treatments within a landscape fuel break network
Source: PLoS One. 2024 Dec 17;19(12):e0313591. doi: 10.1371/journal.pone.0313591 (PMC11651606; doi:10.1371/journal.pone.0313591)
Supplement: S3 Appendix — (DOCX) [file pone.0313591.s003.docx]

**S3 Appendix. Optimization model solutions**

**Notations**

- FBN: fuel break network
- AFB: total avoided fire area burned
- AWB: total avoided WUI area burned
- OPT: optimization
- RBS: rule-based selection

**Table S3.1**. Solutions from 120 optimization (OPT) models and 120 corresponding rule-based selection (RBS) models. Note: FL represents flame length; In each row, OPT/RBS is the value obtained by dividing the OPT value by the RBS value.

| **Model assumptions** | | | **Objective function value**  **(maximizing AFB, hectares)** | | | **Objective function value**  **(maximizing AWB, hectares)** | | |
| --- | --- | --- | --- | --- | --- | --- | --- | --- |
| **Model Fires** | **Escape FL** | **Budget B** | **OPT** | **RBS** | **OPT/RBS** | **OPT** | **RBS** | **OPT/RBS** |
| 80% | 4 | 0.0 | 0 | 0 |  | 0 | 0 |  |
| 80% | 4 | 0.1 | 308781 | 69223 | 4.5 | 3082 | 345 | 8.9 |
| 80% | 4 | 0.2 | 523001 | 120049 | 4.4 | 4034 | 583 | 6.9 |
| 80% | 4 | 0.3 | 691904 | 232021 | 3.0 | 4544 | 1224 | 3.7 |
| 80% | 4 | 0.4 | 824304 | 285039 | 2.9 | 4814 | 1643 | 2.9 |
| 80% | 4 | 0.5 | 934812 | 368709 | 2.5 | 4938 | 2016 | 2.4 |
| 80% | 4 | 0.6 | 1024948 | 466510 | 2.2 | 4997 | 2592 | 1.9 |
| 80% | 4 | 0.7 | 1099997 | 581837 | 1.9 | 5018 | 2968 | 1.7 |
| 80% | 4 | 0.8 | 1156108 | 721154 | 1.6 | 5025 | 3592 | 1.4 |
| 80% | 4 | 0.9 | 1190909 | 906021 | 1.3 | 5025 | 4229 | 1.2 |
| 80% | 4 | 1.0 | 1203135 | 1203135 | 1.0 | 5025 | 5012 | 1.0 |
| 80% | 8 | 0.0 | 0 | 0 |  | 0 | 0 |  |
| 80% | 8 | 0.1 | 1272088 | 205844 | 6.2 | 8398 | 579 | 14.5 |
| 80% | 8 | 0.2 | 2100568 | 357244 | 5.9 | 11792 | 1140 | 10.3 |
| 80% | 8 | 0.3 | 2733650 | 771062 | 3.5 | 14046 | 2515 | 5.6 |
| 80% | 8 | 0.4 | 3263931 | 930719 | 3.5 | 15527 | 3265 | 4.8 |
| 80% | 8 | 0.5 | 3736069 | 1209307 | 3.1 | 16302 | 4134 | 3.9 |
| 80% | 8 | 0.6 | 4162643 | 1587902 | 2.6 | 16686 | 5814 | 2.9 |
| 80% | 8 | 0.7 | 4523378 | 2008825 | 2.3 | 16848 | 7263 | 2.3 |
| 80% | 8 | 0.8 | 4807734 | 2621628 | 1.8 | 16923 | 9651 | 1.8 |
| 80% | 8 | 0.9 | 4974650 | 3583666 | 1.4 | 16945 | 12432 | 1.4 |
| 80% | 8 | 1.0 | 5015541 | 5015541 | 1.0 | 16946 | 16918 | 1.0 |
| 80% | INF | 0.0 | 0 | 0 |  | 0 | 0 |  |
| 80% | INF | 0.1 | 1322297 | 210968 | 6.3 | 8719 | 581 | 15.0 |
| 80% | INF | 0.2 | 2184006 | 364941 | 6.0 | 12343 | 1143 | 10.8 |
| 80% | INF | 0.3 | 2846086 | 791386 | 3.6 | 14669 | 2546 | 5.8 |
| 80% | INF | 0.4 | 3400285 | 958906 | 3.5 | 16235 | 3345 | 4.9 |
| 80% | INF | 0.5 | 3884199 | 1245736 | 3.1 | 17048 | 4236 | 4.0 |
| 80% | INF | 0.6 | 4326799 | 1639675 | 2.6 | 17458 | 6006 | 2.9 |
| 80% | INF | 0.7 | 4706352 | 2074365 | 2.3 | 17624 | 7543 | 2.3 |
| 80% | INF | 0.8 | 5000103 | 2707301 | 1.8 | 17701 | 10024 | 1.8 |
| 80% | INF | 0.9 | 5172212 | 3704152 | 1.4 | 17726 | 12932 | 1.4 |
| 80% | INF | 1.0 | 5213913 | 5213913 | 1.0 | 17727 | 17699 | 1.0 |
| 100% | 4 | 0.0 | 0 | 0 |  | 0 | 0 |  |
| 100% | 4 | 0.1 | 947874 | 112832 | 8.4 | 6429 | 685 | 9.4 |
| 100% | 4 | 0.2 | 1378283 | 164797 | 8.4 | 8455 | 1035 | 8.2 |
| 100% | 4 | 0.3 | 1716780 | 349659 | 4.9 | 9462 | 2637 | 3.6 |
| 100% | 4 | 0.4 | 1968604 | 451775 | 4.4 | 10041 | 3314 | 3.0 |
| 100% | 4 | 0.5 | 2147308 | 604443 | 3.6 | 10379 | 3899 | 2.7 |
| 100% | 4 | 0.6 | 2286490 | 769175 | 3.0 | 10515 | 5021 | 2.1 |
| 100% | 4 | 0.7 | 2393266 | 966188 | 2.5 | 10573 | 5810 | 1.8 |
| 100% | 4 | 0.8 | 2471246 | 1351050 | 1.8 | 10589 | 7152 | 1.5 |
| 100% | 4 | 0.9 | 2517262 | 1769224 | 1.4 | 10591 | 8313 | 1.3 |
| 100% | 4 | 1.0 | 2531917 | 2531917 | 1.0 | 10591 | 10578 | 1.0 |
| 100% | 8 | 0.0 | 0 | 0 |  | 0 | 0 |  |
| 100% | 8 | 0.1 | 5907606 | 346804 | 17.0 | 26367 | 1111 | 23.7 |
| 100% | 8 | 0.2 | 9529844 | 594768 | 16.0 | 42266 | 2109 | 20.0 |
| 100% | 8 | 0.3 | 12311237 | 1774626 | 6.9 | 53794 | 7828 | 6.9 |
| 100% | 8 | 0.4 | 14750813 | 2181829 | 6.8 | 63115 | 9411 | 6.7 |
| 100% | 8 | 0.5 | 16711809 | 2806294 | 6.0 | 69543 | 11318 | 6.1 |
| 100% | 8 | 0.6 | 18542275 | 3554794 | 5.2 | 74427 | 17185 | 4.3 |
| 100% | 8 | 0.7 | 20131625 | 4838998 | 4.2 | 77283 | 21224 | 3.6 |
| 100% | 8 | 0.8 | 21688731 | 6830873 | 3.2 | 79190 | 28192 | 2.8 |
| 100% | 8 | 0.9 | 22640254 | 10764282 | 2.1 | 80276 | 43954 | 1.8 |
| 100% | 8 | 1.0 | 22867788 | 22867788 | 1.0 | 80479 | 80434 | 1.0 |
| 100% | INF | 0.0 | 0 | 0 |  | 0 | 0 |  |
| 100% | INF | 0.1 | 6980426 | 358787 | 19.5 | 31533 | 1125 | 28.0 |
| 100% | INF | 0.2 | 11083292 | 622129 | 17.8 | 50052 | 2156 | 23.2 |
| 100% | INF | 0.3 | 14375597 | 1891500 | 7.6 | 64626 | 8292 | 7.8 |
| 100% | INF | 0.4 | 17182881 | 2319421 | 7.4 | 75650 | 9988 | 7.6 |
| 100% | INF | 0.5 | 19419574 | 2986732 | 6.5 | 83520 | 11957 | 7.0 |
| 100% | INF | 0.6 | 21418200 | 3774003 | 5.7 | 89643 | 18221 | 4.9 |
| 100% | INF | 0.7 | 23264125 | 5134747 | 4.5 | 93309 | 22737 | 4.1 |
| 100% | INF | 0.8 | 25117661 | 7286325 | 3.4 | 95680 | 30969 | 3.1 |
| 100% | INF | 0.9 | 26331070 | 11653189 | 2.3 | 97326 | 49970 | 1.9 |
| 100% | INF | 1.0 | 26698187 | 26698187 | 1.0 | 97749 | 97707 | 1.0 |


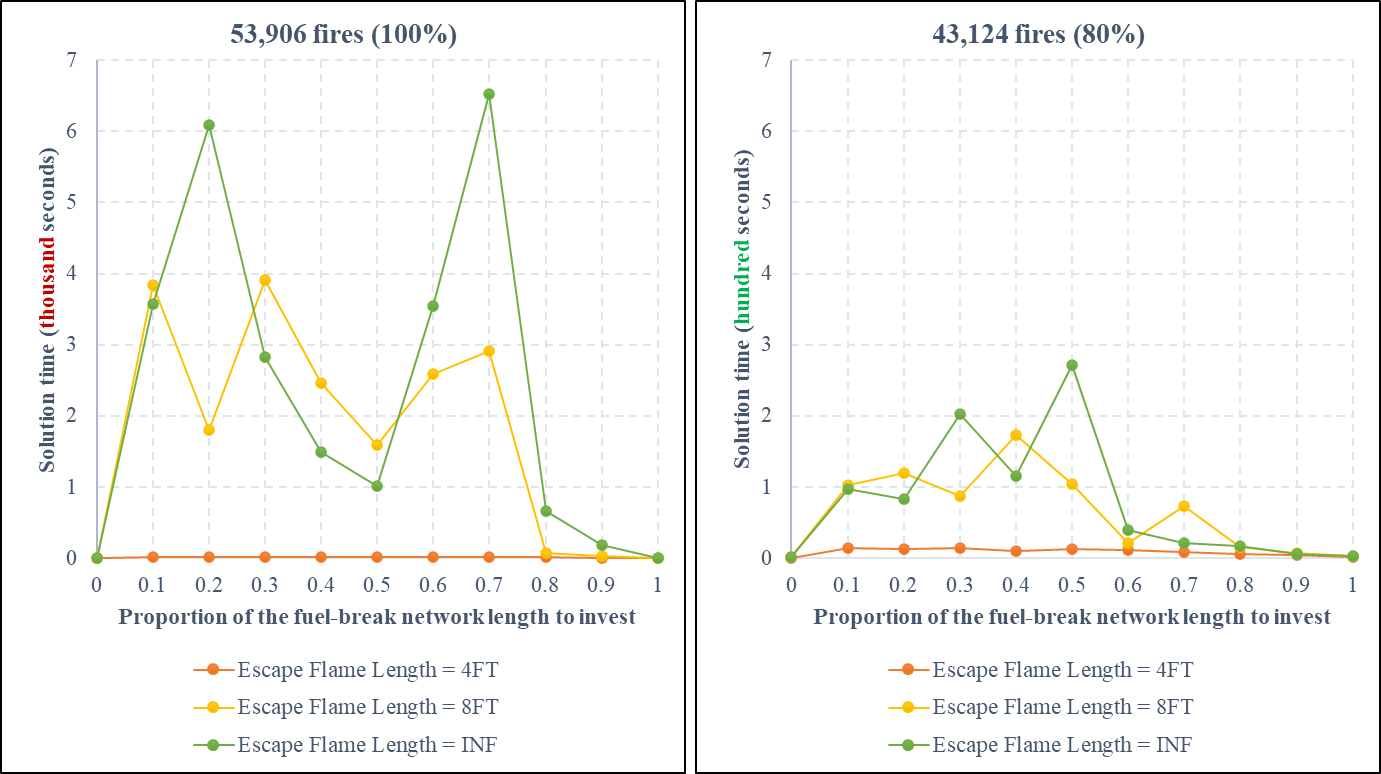


**B**

**A**


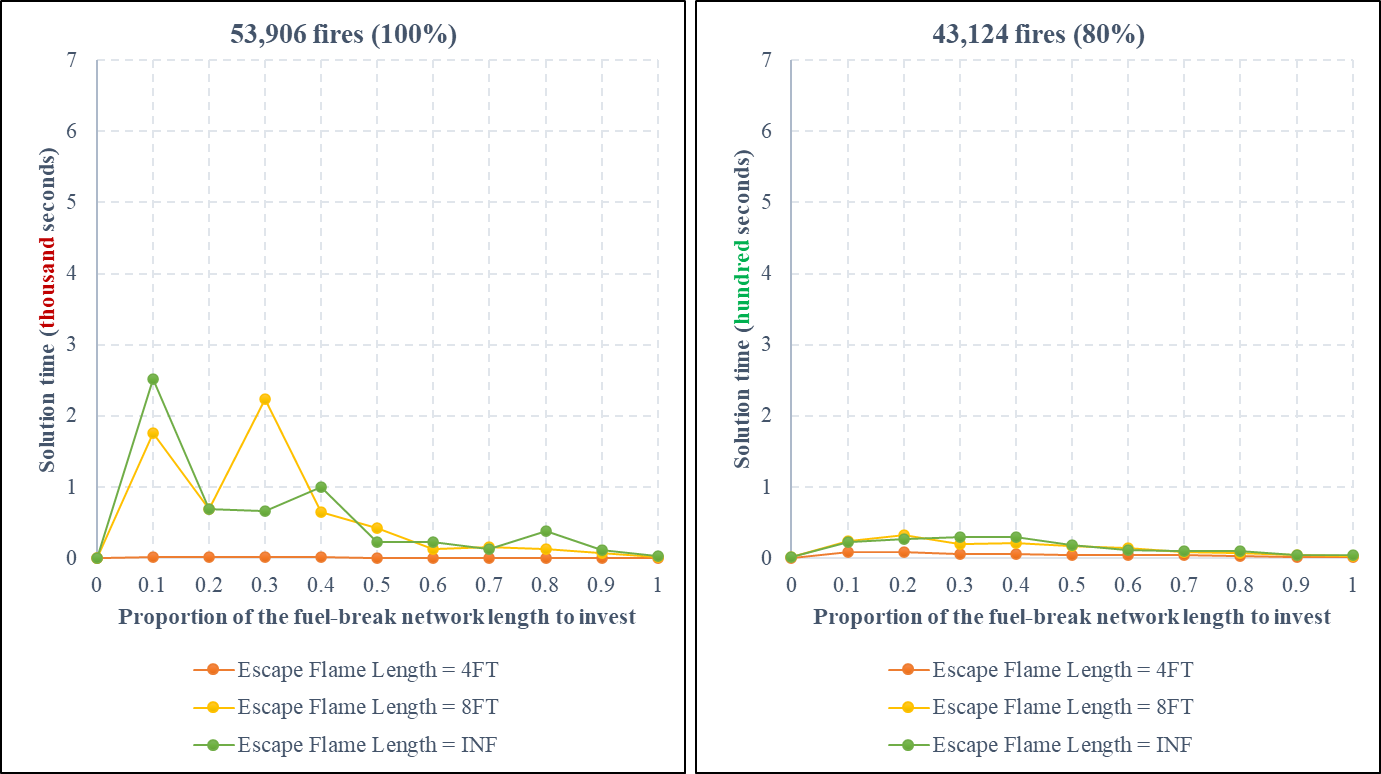


**D**

**C**

**Fig S3.1.** CPLEX solution times of the OPT models. **A**: Maximizing AFB, all fires . **B**: Maximizing AFB, no large fires. **C**: Maximizing AWB, all fires. **D**: Maximizing AWB, no large fires. Note that the time unit is “thousand seconds” in **A** and **C**, while it is “hundred seconds” in **B** and **D**.

**Fig S3.2.** Spatial locations of the fuel breaks selected by the OPT models under the scenario of “maximizing AFB, all fires”. In each map, selected fuel breaks are highlighted as red line-segments; the legend at the bottom left corner includes the percentile of fires to model (100%), the escape flame length (4 FT, 8 FT, or INF), and the budget limit (0.1 to 0.9) separated by the underscore; the legend at the top includes the number of selected breaks and their total length. World Hillshade is used in each map’s background (Sources: Esri, Airbus DS, USGS, NGA, NASA, CGIAR, N Robinson, NCEAS, NLS, OS, NMA, Geodatastyrelsen, Rijkswaterstaat, GSA, Geoland, FEMA, Intermap, and the GIS User Community).

**Fig S3.3.** Spatial locations of the fuel breaks selected by the OPT models under the scenario of “maximizing AFB, no large fires”. In each map, selected fuel breaks are highlighted as red line-segments; the legend at the bottom left corner includes the percentile of fires to model (80%), the escape flame length (4 FT, 8 FT, or INF), and the budget limit (0.1 to 0.9) separated by the underscore; the legend at the top includes the number of selected breaks and their total length. World Hillshade is used in each map’s background (Sources: Esri, Airbus DS, USGS, NGA, NASA, CGIAR, N Robinson, NCEAS, NLS, OS, NMA, Geodatastyrelsen, Rijkswaterstaat, GSA, Geoland, FEMA, Intermap, and the GIS User Community).

**Fig S3.4.** Spatial locations of the fuel breaks selected by the OPT models under the scenario of “maximizing AWB, all fires”. In each map, selected fuel breaks are highlighted as red line-segments; the legend at the bottom left corner includes the percentile of fires to model (100%), the escape flame length (4 FT, 8 FT, or INF), and the budget limit (0.1 to 0.9) separated by the underscore; the legend at the top includes the number of selected breaks and their total length. World Hillshade is used in each map’s background (Sources: Esri, Airbus DS, USGS, NGA, NASA, CGIAR, N Robinson, NCEAS, NLS, OS, NMA, Geodatastyrelsen, Rijkswaterstaat, GSA, Geoland, FEMA, Intermap, and the GIS User Community).

**Fig S3.5.** Spatial locations of the fuel breaks selected by the OPT models under the scenario of “maximizing AWB, no large fires”. In each map, selected fuel breaks are highlighted as red line-segments; the legend at the bottom left corner includes the percentile of fires to model (80%), the escape flame length (4 FT, 8 FT, or INF), and the budget limit (0.1 to 0.9) separated by the underscore; the legend at the top includes the number of selected breaks and their total length. World Hillshade is used in each map’s background (Sources: Esri, Airbus DS, USGS, NGA, NASA, CGIAR, N Robinson, NCEAS, NLS, OS, NMA, Geodatastyrelsen, Rijkswaterstaat, GSA, Geoland, FEMA, Intermap, and the GIS User Community).
