# Supplementary material for: An optimization model to prioritize fuel treatments within a landscape fuel break network
Source: PLoS One. 2024 Dec 17;19(12):e0313591. doi: 10.1371/journal.pone.0313591 (PMC11651606; doi:10.1371/journal.pone.0313591)
Supplement: S4 Appendix — (DOCX) [file pone.0313591.s004.docx]

**S4 Appendix. Modification of the optimization model to maximize the net value**

*Maximize:*$\sum_{j} G_{j}y_{j}-\sum_{j} L_{j}y_{j}-\sum_{i} C_{i}^{F}x_{i}-\sum_{i,j} D_{i,j}^{F}$ (D1)

*Subject to:*

$\sum_{i} C_{i}^{F}x_{i}\leq B$ (D2)

$y_{j}\leq\frac{1}{M_{j}}\sum_{i\in S_{j}^{F}\neq\emptyset} x_{i}$ $\forall j$ (D3)

$y_{j}-\sum_{i\in S_{j}\neq\emptyset} x_{i}\geq\left( 1-M_{j} \right)$ $\forall j$ (D4)

*Where:*

$G_{j}$ is the value protected within the unburned footprint area of fire $j$.

$L_{j}$ is the value lost within burned footprint area of fire $j$.

$C_{i}^{F}$ is the cost for treating in the fuel break $i$ up to a level that can support effective fire suppression under the user-defined condition $F$.

$D_{i,j}^{F}$ is the cost for fire suppression along the fuel break $i$ to respond to fire $j$.

For the definitions of other notations, see the main manuscript.

The modified model presented here differs from the original model outlined in the main manuscript primarily in equations (D1) and (D4). Equation (D1) maximizes the net value by considering benefits from protected land areas, losses due to burning, and costs from both fuel treatment and suppression activities. Notably, a unique feature of the original model lies in the collaborative nature of objective function (1) and equation (3), which effectively captures the coordination of treated fuel breaks for fire containment. However, in the modified model, the collaboration between the corresponding equations (D1) and (D3) no longer work. Therefore, equation (D4) is added to ensure the coordination of treated fuel breaks can be evaluated correctly.
